# Diagnostic performance of left ventricular strain for predicting physiologically significant coronary artery disease

**DOI:** 10.1016/j.ijcrp.2025.200503

**Published:** 2025-09-04

**Authors:** Pratya Rawangban, Anusith Tunhasiriwet, Rawish Wimolwattanaphan, Chanwit Wuttichaipradit, Piyoros Lertsanguansinchai

**Affiliations:** a Chulabhorn Royal Academy Hospital, Bangkok, Thailand; b Division of Cardiovascular Medicine, Department of Medicine, Faculty of Medicine, Chulalongkorn University, Bangkok, Thailand; c Cardiac Center, King Chulalongkorn Memorial Hospital, The Thai Red Cross Society, Bangkok, Thailand

**Keywords:** Physiologically significant coronary artery disease, Fractional flow reserve, FFR, Non-hyperemic pressure ratios, NHPRs, Instantaneous wave free ratio, iFr, Left ventricular global longitudinal strain, LV GLS

## Abstract

**Background:**

Coronary artery disease (CAD) remains a leading cause of morbidity and mortality worldwide. While invasive coronary angiography is the gold standard for diagnosing obstructive CAD, contemporary guidelines advocate for initial evaluation using resting transthoracic echocardiography. The role of left ventricular global longitudinal strain (LV GLS) as a screening tool in the diagnosis of obstructive CAD remains unclear.

**Objective:**

To evaluate the diagnostic utility of LV GLS in predicting physiologically significant CAD as confirmed by intracoronary physiological tests such as fractional flow reserve (FFR) and/or non-hyperemic pressure ratios (NHPRs).

**Methods:**

We conducted a retrospective cohort study at the Cardiac Center, Chulabhorn Hospital, Thailand, enrolling patients with suspected CAD who underwent coronary angiography with FFR and/or NHPRs between August 2018 and September 2024. Resting echocardiograms were reevaluated for LV GLS. Receiver operating characteristic (ROC) analysis was performed to assess the predictive value of LV GLS for physiologically significant CAD. A multivariate model incorporating LV GLS patterns and clinical parameters was also developed.

**Results:**

Of the 207 patients analyzed, 99 (47 %) had positive physiological test results (FFR ≤0.80 and/or NHPR ≤0.89). The average LV GLS was lower in the physiologically positive group (−15.6 %) compared to the negative group (−16.8 %), though this difference was not statistically significant. ROC analysis of average LV GLS yielded an area under the curve (AUC) of 0.56 (95 % CI: 0.48–0.64, p = 0.130). However, the ischemic Bull's-eye pattern derived from LV GLS demonstrated high sensitivity (93 %) and negative predictive value (87 %). Multivariate analysis identified central aortic pulse pressure (OR 1.02, 95 % CI = 1.00–1.04, p = 0.042) and the ischemic Bull's-eye pattern (OR 16.33, 95 % CI = 5.16–51.72, p < 0.001) as independent predictors of physiologically significant CAD. The combined model achieved an AUC of 0.76, outperforming both the average LV GLS alone and 2024 ESC clinical risk factor-based likelihood.

**Conclusions:**

While the average LV GLS is not a robust predictor of physiologically significant CAD, the ischemic Bull's-eye pattern derived from LV GLS offers high sensitivity and negative predictive value. When combined with central aortic pulse pressure, this approach enhances the diagnostic accuracy for physiologically significant CAD. These findings support the integration of ischemic Bull's-eye patterns derived from LV GLS and central aortic pulse pressure into initial CAD screening protocols.

## 1 Background

Coronary artery disease (CAD) is a common condition that frequently prompts patients to seek medical attention. In Thailand, the prevalence of CAD has increased, currently affecting 1.89 % of the population aged 60 years and older [1].CAD is the main cause of myocardial infarction, which is associated with a high mortality rate, reaching as high as 14 % [2]. Patient with CAD can manifest in different ways [3], depending on the progression and mechanisms underlying the disease. Acute coronary syndrome (ACS) is one of the common presentations, which occurs when atherosclerotic plaque ruptures or erodes. ACS typically develops suddenly and is associated with more severe symptoms, consequently most patients will undergo coronary angiography to determine if obstructive CAD is present [4]. On the other hand, some patients with CAD may experience chronic coronary syndrome (CCS), that tend to develop more gradually over time. The underlying cause is usually a fixed obstruction in the coronary arteries [5]. As a result, symptoms may develop during physical exertion, when there is a mismatch between oxygen demand and supply. In addition to this, others mechanism, such as microvascular dysfunction can also contribute to CCS [6]. Due to the gradual onset of symptoms, patients with CCS are typically first evaluated with non-invasive tests. The 2024 ESC guidelines recommended that patients suspected of having CCS undergo diagnostic modalities such as stress echocardiography, stress cardiac magnetic resonance imaging (CMR), positron emission tomography (PET), or single photon emission computed tomography (SPECT), depending on their pre-test probability of the disease, the expertise available at the local center, and any contraindications for these tests [7]. However, resting transthoracic echocardiography (TTE), which is a basic and readily accessible test, is recommended as a Class I indication as an initial evaluation [7].

According to the recent ESC guidelines, a resting transthoracic echocardiogram is recommended to evaluate left ventricular systolic function, diastolic function, RV function [7]. It also helps assess regional wall motion abnormalities, which are indicators of obstructive CAD. If the risk factor-weighted clinical likelihood of obstructive CAD exceeds 85 %, patients should undergo invasive coronary angiography (ICA) [7]. Due to the invasive in nature and limited availability of ICA, the procedure is reserved for patients with severe symptoms refractory to antianginal medications, those experiencing angina or dyspnea at low level of exertion, individual with left ventricular dysfunction suggesting extensive obstructive CAD, or when non-invasive testing indicates a high event risk [3,7]. In clinical practice it would be ideal to identify simple, noninvasive diagnostic tools for detect regional or subclinical left ventricular systolic dysfunction before proceeding with more specialized tests.

Left ventricular global longitudinal strain (LV GLS) is the novel imaging technique that utilizes speckle tracking echocardiography to measure myocardial length at end systole(MLs) and diastole(MLd), and then calculates global longitudinal strain using the following formula: GLS(%) = (MLs - MLd)/MLd [8]. The more negative GLS value indicates better systolic function, with an expected normal value typically around −18 % [[8], [9], [10]]. LV GLS, measured by speckle tracking, is increasingly used in clinical practice, particularly within the field of cardio-oncology [11], to evaluate cancer therapy-related cardiac dysfunction. While LV GLS is not yet a standard imaging tool for coronary artery disease. A study by Norum IB et al. demonstrated that LV GLS can predict obstructive CAD (defined as >50 % coronary artery stenosis) with modest sensitivity and specificity [12]. Using a cut point off range of −17.4 % to −19.7 %, the sensitivity of LV GLS ranged from of 51 %–81 %, and specificity ranged from 58 % to 81 % [12].

Currently the ESC guidelines for the management of chronic coronary syndrome recommend the use of physiologic tests, such as fractional flow reserve (FFR) and/or non hyperemic pressure ratio like the instantaneous wave free ratio (iFR), to assess the functional severity of intermediate coronary stenosis [7]. These physiological tests are considered the current gold standard for determining whether a patient has significant coronary artery stenosis [7,13,14]. A study by Ozawa et al. demonstrated that LV GLS can predict physiologically significant coronary artery disease when using these physiologic tests as the gold standard [15]. They reported that, when using a cut-off of −12 % for LV GLS, the sensitivity was 70.4 % and the specificity was 57.9 % [15]. A key limitation of this study was the small sample size, that lead to an underestimation of the diagnostic performance of LV GLS.

## 2 Objective

We plan to conduct a clinical study to evaluate the diagnostic performance of left ventricular global longitudinal strain (LV GLS) in predicting physiologically significant coronary artery disease (CAD), confirmed by intracoronary physiological tests such as fractional flow reserve (FFR) and/or non-hyperemic pressure ratios (NHPRs), as the gold standard.

## 3 Method

### 3.1 Study design

We conducted a retrospective, cohort study at the Cardiac Center, Chulabhorn Hospital, Thailand. The enrollment criteria included all consecutive patients who presented with symptoms suspected of CAD and underwent invasive coronary angiography, along with physiological testing using either fractional flow reserve (FFR) or non-hyperemic pressure ratio (NHPRs). Resting echocardiogram, collected prior to coronary angiography, was used to re-evaluate LV GLS. The protocol was approved by the Chulabhorn Royal Academy Ethics Committee (EC 116/2566) before data collection commenced.

### 3.2 Patients

The eligibility criteria included patients with signs or symptoms of coronary artery disease, either chronic coronary syndrome or acute coronary syndrome, who underwent coronary angiography prior to cardiac surgery. This approach aimed to evaluate the characteristics and generalizability of the LV GLS test. All patients had a pre-catheterization resting echocardiogram within 6 months. Patients were excluded from the study if they had previously undergone coronary intervention in the same vascular territory or had a history of coronary artery bypass graft (CABG) surgery. Patients with severe valvular heart disease or those with significantly impaired left ventricular systolic function (defined as a left ventricular ejection fraction [LVEF] of less than 40 %) were also excluded. Additionally, patients with ST-elevation myocardial infarction (STEMI) involving the culprit vessel were excluded, as this condition may affect LV GLS measurements independently of obstructive coronary artery disease.

### 3.3 Invasive intracoronary physiological tests (gold standard)

We considered invasive coronary physiological tests, including fractional flow reserve (FFR) and/or non-hyperemic pressure ratios as the gold standard (NHPRs). FFR is a diagnostic technique used to assess the functional significance of coronary artery stenosis by measuring the pressure difference across a stenosis during coronary artery catheterization, determining whether the narrowing is causing a significant reduction in blood flow leading to ischemia [16]. An FFR value ≤ 0.80 is considered a positive result [7,16]. Non-hyperemic pressure ratios such as instantaneous wave-free ratio (iFR), similar to FFR, measures the functional significance of coronary stenosis but does not require vasodilators like adenosine to induce hyperemia [17]. iFR is measured during the wave-free period of the cardiac cycle and does not require pharmacologic stress [17]. An iFR value ≤ 0.89 is considered a positive result [7,17].

According to current guidelines, physiological tests are recommended for patients with intermediate coronary stenosis, typically defined as 40–90 % for non-left main disease and 40–70 % for left main disease [7]. For patients with stenosis exceeding these thresholds (i.e., >90 % for non-left main disease or >70 % for left main disease), the stenosis is considered obstructive, and physiological testing is generally not required. Patients with stenosis below the lower limit of these ranges are generally considered to have non-obstructive CAD [7].

### 3.4 Echocardiography

A resting echocardiogram was performed on all patients within 6 months prior to coronary angiography. Parameters collected included left ventricular dimensions (end-diastolic [LVIDd] and end-systolic [LVIDs]), interventricular septum thickness at end-diastole (IVSd), left ventricular posterior wall thickness at end-diastole (LVPWd), and left ventricular systolic function (LVEF). Regional wall motion abnormalities were assessed by visual evaluation. Diastolic function, right ventricular function, and left atrial volume index were also recorded.

### 3.5 Left ventricular global longitudinal strain

Pre-catheterization resting echocardiograms were re-evaluated using TOMTEC software (Philips). Speckle tracking technology was used to measure myocardial length at end-systole (MLs) and end-diastole (MLd), with global longitudinal strain (GLS) calculated using the formula: GLS(%) = (MLs - MLd)/MLd [[8], [9], [10]].

A more negative GLS value indicates better systolic function, with a typical normal value around −18 % [[8], [9], [10]].

The parameters derived from this calculation, including average LV GLS and regional LV GLS for each vascular territory, were used to evaluate diagnostic performance.

### 3.6 Regional left ventricular strain according to coronary artery territories

**Fig. 1:**
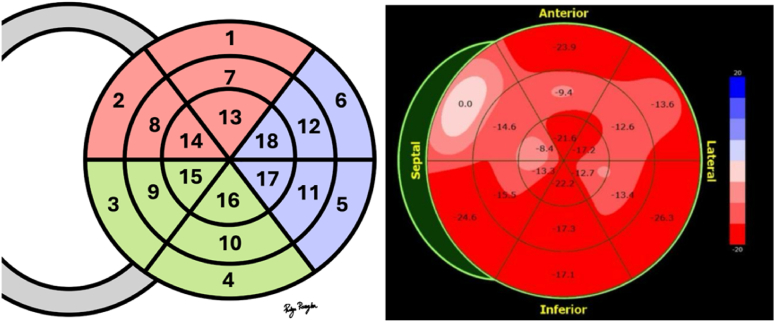
Shows myocardial segmentation using the 18-segment model (left side) and an example of a patient with an abnormal Bull's-eye pattern on LAD territory (right side). Adapted from Lang et al., 2015, ASE Guidelines for Cardiac Chamber Quantification [8].

We defined regional left ventricular (LV) global longitudinal strain (GLS) based on myocardial segmentation using the 18-segment model. Regional LV GLS for the left anterior descending artery (LAD) included segments 1, 2, 7, 8, 13, and 14 (shown in red); for the left circumflex artery (LCx), it included segments 5, 6, 11, 12, 17, and 18 (shown in blue); and for the right coronary artery (RCA), it included segments 3, 4, 9, 10, 15, and 16 (shown in green), as demonstrated in Fig. 1.

### 3.7 Ischemic Bull's-eye pattern derived from the LV GLS test

We defined an ischemic Bull's-eye pattern based on the presence of more than two consecutive segments with an LV GLS of less than −15 % in each regional territory of the coronary vessels. If such abnormalities were identified in any regional territory, the test was considered abnormal. For example, the Bull's-eye pattern derived from LV GLS in Fig. 1 (right side) demonstrates an ischemic Bull's-eye pattern in the LAD territory due to a decrease in LV GLS to less than −15 % in more than two consecutive segments in the LAD territory.

### 3.8 Statistical analysis

Based on the study by Norum et al. [12], which reported a sensitivity of 81 % for GLS testing, and the prevalence of obstructive CAD (14.2 %) from Suwatanaviroj et al. [18], the sample size was calculated to require at least 115 patients. Categorical variables were analyzed using the Chi-square test or Fisher's exact test, while continuous variables were analyzed using the Mann-Whitney *U* test. To evaluate the diagnostic performance of LV GLS, receiver operating characteristic (ROC) analysis was performed.

Additionally, we aimed to assess the diagnostic performance of the ischemic Bull's-eye pattern, which is commonly used in clinical practice, though it lacks strong supporting evidence. Sensitivity and specificity were calculated to evaluate its diagnostic utility.

Furthermore, univariate and multivariate logistic regression analyses were conducted to identify factors associated with physiologically significant coronary artery disease. Factors with a p-value of less than 0.2 in the univariate analysis were included in the multivariate analysis, and results are presented with adjusted odds ratios and 95 % confidence intervals (CIs). A p-value of less than 0.05 was considered statistically significant.

Inter-observer and intra-observer variability were also tested using the Student's t-test and Bland-Altman analysis for LV GLS measurement, and Cohen's Kappa value for the ischemic Bull's-eye pattern for 20 consecutive patients randomly selected from the study population.

## 4 Results

### 4.1 Population

All consecutive patients who underwent coronary angiography with physiological testing using either fractional flow reserve (FFR) or non-hyperemic pressure ratio (NHPRs) were eligible for inclusion in the clinical trial. From August 2018 to September 2024, a total of 260 patients were screened. After applying the appropriate

**Fig. 2:**
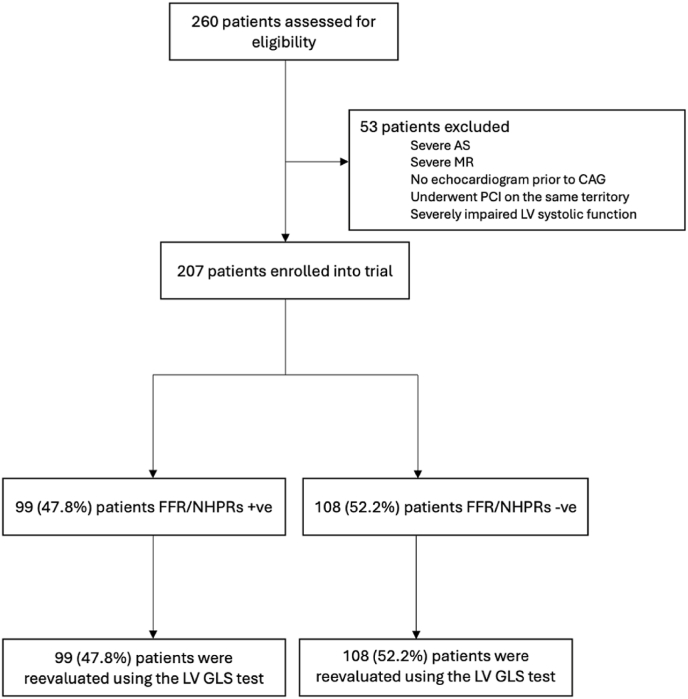
Demonstrates the consort diagram of patients in clinical trial.

Inclusion and exclusion criteria, 207 participants were eligible for analysis. The majority of patients who underwent coronary angiography with physiological testing had chronic coronary syndrome, with only a small number diagnosed with acute coronary syndrome (ACS). Of the 207 patients, 99 (47 %) were classified as having positive physiological test results (FFR ≤0.80 and/or iFR ≤0.89), while the remaining patients had negative results (Fig. 2).

The mean age of the participants was approximately 66 years, and 35 % were women. The clinical characteristics of the two groups (physiologically significant CAD and insignificant CAD) were well balanced in terms of risk factors, previous medical conditions and laboratory tests (Table 1).

**Table 1:** Demographic data and laboratory tests.

Variables	Total	Physiologically significant CAD		P-value
		FFR/NHPRs + (n = 99)	FFR/NHPRs - (n = 108)	
**Sex**				0.325
Male – no./total no. (%)	133 (64.25)	67 (67.68)	66 (61.11)	
Female – no./total no. (%)	74 (35.75)	32 (32.32)	42 (38.89)	
**Age** – yr	66.87 ± 10.28	67.53 ± 10.95	66.27 ± 9.63	0.381
**BMI** (IQR) – Kg/m^2^	24.09 (22.35–26.50)	23.63 (21.67–26.63)	24.60 (22.66–26.67)	0.095
**SBP** (IQR) – mmHg	149 (136–167)	149 (136–168)	149 (136.5–167)	0.666
**DBP** (IQR) – mmHg	80 (73–92)	79 (69–89)	82.5 (74–92.5)	0.035
**Heart rate** (IQR) – beats/min	70 (63–80)	70 (62–80)	69 (64–79)	0.776
**Smoking** – no./total no. (%)	8 (3.86)	6 (6.06)	2 (1.85)	0.156
**Diabetes mellitus** – no./total no. (%)	84 (40.58)	45 (45.45)	39 (36.11)	0.171
**Hypertension** – no./total no. (%)	149 (71.98)	71 (71.72)	78 (72.22)	0.936
**Dyslipidemia** – no./total no. (%)	160 (77.29)	77 (77.78)	83 (76.85)	0.874
**Chronic kidney disease** – no./total no. (%)	20 (9.66)	10 (10.10)	10 (9.26)	0.838
**History of stroke** – no./total no. (%)	13 (6.28)	9 (9.09)	4 (3.70)	0.111
**Malignancy** – no./total no. (%)	7 (3.40)	2 (2.04)	5 (4.63)	0.449
**Chronic obstructive pulmonary disease** – no./total no. (%)	7 (3.38)	5 (5.05)	2 (1.85)	0.263
**Previous CAD** – no./total no. (%)	70 (33.82)	38 (38.38)	32 (29.63)	0.184
**2024 ESC clinical risk score** (IQR) – point	12 (6–21)	17 (6–22)	11 (6–17)	0.070
**BUN** (IQR) – mg/dL		16 (11–20)	16 (13–20)	0.215
**Creatinine** (IQR) – mg/dL		0.97 (0.77–1.17)	0.95 (0.73–1.13)	0.507
**eGFR** (IQR) – mL/min/1.73 m^2^		78 (60–92)	97 (61–91)	0.869
**Hemoglobin** (IQR) – g/dL		13.00 (11.80–14.00)	13.00 (12.1–13.90)	0.580
**Hematocrit** (IQR) – %		39.90 (36.00–42.00)	40.00 (37.35–42.00)	0.357
**White blood cell** (IQR) – cells/μl		6510 (5630–8020)	6785 (5400–8395)	0.501
**Platelet** (x10^3^) (IQR) – platelets/μl		222 (185–278)	224 (189–288)	0.522
**Fasting blood glucose** (IQR) – mg/dL		106 (98–129)	103 (96–116)	0.142
**HbA1C** (IQR) – %		6.20 (5.60–6.90)	6.00 (5.60–6.20)	0.076
**Total cholesterol** (IQR) – mg/dL		146.00 (129.00–170.00)	142.50 (132.00–183.50)	0.815
**HDL** (IQR) – mg/dL		51.00 (42.00–59.00)	52.50 (45.00–61.50)	0.274
**LDL** (IQR) – mg/dL		77.00 (60.00–101.00)	78 (60–104)	0.921

### 4.2 Cardiac catheterization parameters

Most patients had stenosis in the left anterior descending artery (LAD), with the degree of stenosis being 58.11 ± 22.83 % in the physiologically positive group and 48.86 ± 22.81 % in the physiologically negative group (Table 2). The median fractional flow reserve (FFR) was 0.76 (IQR 0.7–0.8) in the physiologically positive group and 0.88 (IQR 0.85–0.92) in the physiologically negative group. Most of the patients in the positive physiological test group underwent percutaneous coronary intervention (PCI), while those in the negative test group primarily received medical therapy.

**Table 2:** Cardiac catheterization parameters.

Variables	Physiologically significant CAD		P-value
	FFR/NHPRs +	FFR/NHPRs -	
**Clinical diagnosis**			0.237
Chronic coronary syndrome – no./total no. (%)	82 (82.83)	98 (90.74)	
STEMI – no./total no. (%)	6 (6.06)	3 (2.78)	
NSTEMI – no./total no. (%)	9 (9.09)	4 (3.70)	
Preoperative CAG – no./total no. (%)	2 (2.02)	3 (2.78)	
Central aortic pulse pressure (IQR) – mmHg	69 (58–86)	64 (53.5–79)	0.038
LM stenosis – %	9.75 ± 18.65	4.44 ± 12.73	0.013
LAD stenosis – %	58.11 ± 22.83	48.86 ± 22.81	<0.001
LCx stenosis – %	44.90 ± 31.90	34.31 ± 29.67	0.013
RCA stenosis – %	36.72 ± 31.22	36.16 ± 30.11	0.983
FFR (IQR)	0.76 (0.70, 0.80)	0.88 (0.85, 0.92)	<0.001
NHPRs (IQR)	0.86 (0.80, 0.89)	0.95 (0.92, 0.97)	<0.001
**Target vessel**			0.004
LAD – no./total no. (%)	73 (73.74)	56 (51.85)	
LCx – no./total no. (%)	12 (12.12)	30 (27.78)	
RCA – no./total no. (%)	14 (14.14)	21 (19.44)	
LM – no./total no. (%)	0 (0.00)	1 (0.93)	
**Management**			<0.001
PCI – no./total no. (%)	64 (64.65)	4 (3.70)	
Medications – no./total no. (%)	26 (26.26)	102 (94.44)	
Plan stage PCI – no./total no. (%)	3 (3.03)	0 (0)	
CABG – no./total no. (%)	6 (6.06)	0 (0)	

LAD, left anterior descending artery; LCx, left circumflex artery; RCA, right coronary artery; LM, left main; PCI, percutaneous coronary intervention; CABG, coronary artery bypass graft.

### 4.3 Echocardiographic parameters

The results from the resting comprehensive echocardiogram are presented in Table 3. Overall, left ventricular (LV) systolic function was preserved in both groups. Regional wall motion abnormalities were absent in most patients. There is no significant difference between the two groups in terms of these abnormalities. Most patients were classified as having grade 1 diastolic dysfunction.

**Table 3:** Echocardiographic parameters.

Variables	Physiologically significant CAD		P-value
	FFR/NHPRs +	FFR/NHPRs -	
LV size			0.464
Normal size – no./total no. (%)	71 (71.72)	78 (72.22)	
Concentric hypertrophy – no./total no. (%)	22 (22.22)	27 (25.00)	
Eccentric hypertrophy – no./total no. (%)	6 (6.06)	3 (2.78)	
LV dimension in end diastole (IQR) – mm	30.00 (25.00, 35.00)	27.00 (25.00, 32.00)	0.125
Interventricular septum thickness in end diastole (IQR) – mm	10.80 (9.00, 12.20)	11.00 (9.40, 12.66)	0.457
LV posterior wall thickness in end diastole (IQR) – mm	10.20 (9.00, 12.25)	10.90 (9.00, 12.00)	0.729
LVEF (IQR) – %	59.00 (50.00, 65.00)	62.00 (54.50, 66.00)	0.094
Regional wall motion abnormality – no./total no. (%)	17 (17.17)	19 (17.59)	0.936
Wall motion score index (IQR)	1.00 (1.00, 1.25)	1.00 (1.00, 1.00)	0.183
Diastolic function			0.727
Normal – no./total no. (%)	16 (16.16)	23 (21.30)	
Grade 1 – no./total no. (%)	62 (62.63)	58 (53.70)	
Grade 2 – no./total no. (%)	11 (11.11)	12 (11.11)	
Grade 3 – no./total no. (%)	2 (2.02)	3 (2.78)	
Indeterminate – no./total no. (%)	8 (8.08)	12 (11.11)	
E/A (IQR)	0.80 (0.69, 0.91)	0.79 (0.66, 1.12)	0.958
Septal E/e' (IQR)	11.35 (14.85, 9.56)	11.00 (9.30, 14.00)	0.408
Lateral E/e' (IQR)	9.00 (7.00, 11.01)	7.90 (6.50, 10.04)	0.048
Average E/e' (IQR)	10.21 (8.00, 12.60)	9.00 (7.70, 11.10)	0.079
TR Vmax (IQR) – m/sec	2.36 (2.02, 2.80)	2.32 (2.09, 2.50)	0.640
TAPSE (IQR) – mm	22.00 (19.00, 23.90)	22.00 (19.00, 24.00)	0.903
TV Lateral S' (IQR) – cm/sec	13.00 (11.50, 14.50)	12.40 (11.00, 14.00)	0.098
Left atrial volume index (IQR) – mL/m^2^	34.07 (27.60, 42.30)	32.90 (23.00, 38.56)	0.146
Average LV GLS (%)	15.60 (11.80, 18.10)	16.80 (14.00, 18.80)	0.115
Regional LV GLS on LAD (%)	15.73 (11.83, 18.53)	15.69 (13.21, 18.21)	0.590
Regional LV GLS on LCx (%)	16.35 (12.32, 18.72)	17.67 (14.68, 20.30)	0.019
Regional LV GLS on RCA (%)	14.98 (10.92, 17.60)	15.90 (12.57, 17.85)	0.207

LV GLS, left ventricular global longitudinal strain; LAD, left anterior descending artery; LCx, left circumflex artery; RCA, right coronary artery.

For the LV GLS test, the results are presented in Table 3. The median average GLS was lower in the physiologically positive group (15.6 %) compared to the negative group (16.8 %), though this difference was not statistically significant. The receiver operating characteristic (ROC) analysis of average LV GLS for predicting physiologically significant coronary artery disease (CAD) yielded an area under the curve (AUC) of 0.56 (95 % CI: 0.48–0.64), which was not statistically significant (p-value = 0.130). Regional LV GLS measurements for the left anterior descending artery (LAD) and right coronary artery (RCA) territories were also not statistically significant. However, regional LV GLS for the left circumflex artery (LCx) territory demonstrated modest diagnostic performance in predicting physiologically significant CAD, with an AUC of 0.59 (95 % CI: 0.51–0.67, p-value = 0.015).

### 4.4 Ischemic Bull's-eye pattern derived from the LV GLS test

**Fig. 3:**
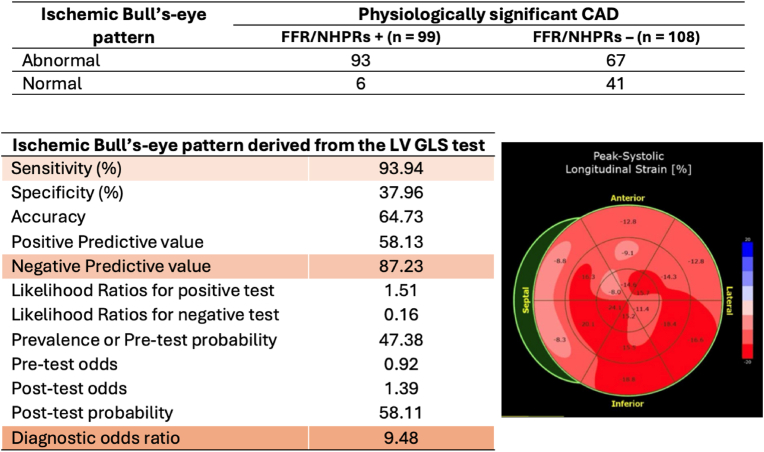
Ischemic Bull's-eye pattern derived from the LV GLS test.

In addition to evaluating the absolute value of LV GLS, we also analyzed abnormalities in the Bull's-eye pattern derived from the LV GLS test. We defined an ischemic Bull's-eye pattern based on the presence of greater than two consecutive segments with an LV GLS less than −15 % in each regional territory of the coronary vessels. If such abnormalities were identified in any regional territory, the test was considered abnormal. The analysis of the ischemic Bull's-eye pattern derived from the LV GLS test demonstrated a high sensitivity of 93 % and a negative predictive value of 87 %. However, the specificity was relatively low, at 38 %. The diagnostic odds ratio for this ischemic Bull's-eye pattern was 9.48 (Fig. 3).

### 4.5 Factors associated with physiologically significant coronary artery disease

Univariate and multivariate logistic regression analyses were performed to identify factors associated with physiologically significant coronary artery disease. Univariate analysis revealed that the HbA1c level, central aortic pulse pressure, history of prior ischemic stroke, previous coronary artery disease, septal E/e’, lateral E/e’, average E/e’, average LV GLS, regional LV GLS in the left circumflex artery territory, and an abnormal Bull's eye pattern on LV GLS were associated with physiologically significant coronary artery disease (Table 4).

**Table 4:** Factors associated with physiologically significant coronary artery disease.

Variables	Crude OR (95 % CI)	P-value	Adjust OR (95 % CI)	P-value
HbA1C level	1.67 (1.07, 2.60)	0.023		
Central aortic pulse pressure	**1.01 (1.00, 1.02)**	**0.155**	**1.02 (1.00, 1.04)**	**0.042**
Old ischemic stroke	2.60 (0.77, 8.73)	0.122		
Previous coronary artery disease	1.48 (0.83, 2.64)	0.184		
Septal E/e’	1.04 (0.99, 1.08)	0.098		
Lateral E/e’	1.07 (1.00, 1.15)	0.061		
Average E/e’	1.07 (1.01, 1.13)	0.033		
Average LV GLS	0.95 (0.89, 1.02)	0.130		
Regional LV GLS on LCx territory	0.93 (0.87, 0.99)	0.015		
Ischemic Bull's eye pattern from LV GLS	**9.49 (3.81, 23.62)**	**<0.001**	**16.33 (5.16, 51.72)**	**<0.001**

LV GLS, left ventricular global longitudinal strain; LCx, left circumflex artery; OR, odds ratio.

**Fig. 4:**
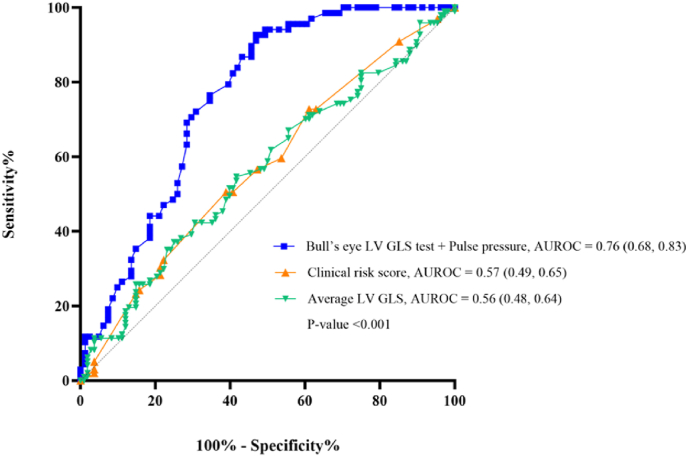
Present diagnostic accuracy of the model combining the ischemic Bull's-eye pattern derived from the LV GLS test and central aortic pulse pressure in predicting physiologically significant CAD, compared with the average LV GLS value alone and the risk factor-weighted clinical likelihood of obstructive CAD.

In the multivariate analysis, the independent predictors of physiologically significant coronary artery disease were central aortic pulse pressure (OR 1.02, 95 % CI = 1.00–1.04, p = 0.042) and an ischemic Bull's eye pattern on LV GLS (OR 16.33, 95 % CI = 5.16–51.72, p < 0.001) (Table 4). Fig. 4 demonstrates diagnostic performance of central aortic pulse pressure and the presence of the ischemic Bull's eye pattern in predicting physiologically significant CAD. When compared with the risk factor-weighted clinical likelihood of obstructive coronary artery disease as outlined in the 2024 ESC guidelines, the diagnostic performance of this model demonstrated superior accuracy.

### 4.6 Intra-observer and inter-observer variability

An analysis of intra-observer and inter-observer variability of LV GLS and the ischemic Bull's eye pattern were performed in 20 test-retest studies. There was no statistically significant difference for any component of LV GLS. The inter-observer correlation for LV GLS was 0.93 (apical 4-chamber view), 0.81 (apical 3-chamber view), 0.94 (apical 2-chamber view), and 0.95 (average LV GLS). The intra-observer correlation for LV GLS was 0.92 (apical 4-chamber view), 0.74 (apical 3-chamber view), 0.89 (apical 2-chamber view), and 0.97 (average LV GLS).

Furthermore, there was near perfect agreement for inter-observer correlation in the interpretation of the ischemic Bull's eye pattern, confirmed by Cohen's Kappa values of 0.85.

## 5 Discussion

We conducted a retrospective cohort study at the Cardiac Center, Chulabhorn Hospital, from August 2018 to September 2024. A total of 207 participants, who were diagnosed with suspected coronary artery disease and underwent coronary angiography with intracoronary physiological testing, were eligible for the final analysis. Of these, 99 (47 %) patients were classified as having a physiologically significant test (FFR ≤0.80 and/or NHPRs ≤0.89).

Patients with positive physiological test results tended to have lower average left ventricular (LV) global longitudinal strain (GLS), though this difference was not statistically significant. The receiver operating characteristic (ROC) curve for average LV GLS yielded an area under the curve (AUC) of 0.56 (95 % CI: 0.48–0.64), which did not reach statistical significance (p = 0.130). This indicates that average LV GLS values cannot reliably predict obstructive coronary artery disease (CAD) as confirmed by invasive physiological testing. However, when examining regional LV GLS, the left circumflex artery territory was the only region where regional GLS demonstrated modest diagnostic accuracy in predicting physiologically significant CAD, with an AUC of 0.59 (95 % CI: 0.51–0.67) and a p-value of 0.015. The primary limitation of using absolute LV GLS values, both global and regional, to predict obstructive CAD is that these values are derived from the average of all LV GLS segments. Normal segments can have higher values, which may compensate for segments affected by ischemia or infarction [19]. As a result, the average LV GLS may not differ significantly between patients with obstructive CAD and those with non-obstructive CAD. Regional wall motion abnormalities, which derived from visual assessment, were also not effective in diagnosing obstructive CAD, as there was no significant difference between patients with obstructive and non-obstructive CAD, as confirmed by invasive intracoronary testing.

Our results are consistent with the study by Ozawa et al. [15], which found that average LV GLS is not a reliable diagnostic tool for predicting obstructive coronary artery disease (CAD). However, when utilizing the ischemic Bull's-eye pattern derived from the LV GLS test, we observed high sensitivity, negative predictive value, and a diagnostic odds ratio of 9.48, albeit with low specificity. This suggests that the ischemic Bull's-eye pattern can serve as a valuable screening tool for predicting obstructive CAD, particularly when used in conjunction with other diagnostic tests. Due to its high sensitivity and negative predictive value, a normal LV GLS along with an unremarkable Bull's-eye pattern may indicate that a patient is unlikely to have significant CAD. Conversely, a positive result would warrant further diagnostic evaluation to confirm the presence of obstructive CAD.

Furthermore, univariate and multivariate logistic regression analyses were conducted to identify factors associated with physiologically significant coronary artery disease. In the multivariate analysis, the independent predictors of physiologically significant CAD were central aortic pulse pressure (OR 1.02, 95 % CI = 1.00–1.04, p = 0.042) and an ischemic Bull's eye pattern on LV GLS (OR 16.33, 95 % CI = 5.16–51.72, p < 0.001). The widening of central aortic pulse pressure may reflect arterial stiffness (stiff aorta), which has been associated with coronary artery disease [20,21]. The central aortic pulse pressure alone showed modest accuracy in diagnosing physiologically significant CAD, with an AUC of 0.58 and a best cutoff point of 65 mmHg. When this model was used to predict physiologically significant CAD, it yielded an AUC of 0.76, demonstrating greater accuracy than the risk factor-weighted clinical likelihood of obstructive coronary artery disease, as shown in Fig. 4. These findings suggest that incorporating this clinical factor, along with the ischemic Bull's eye pattern on LV GLS, may serve as an initial diagnostic screening tool for patients suspected of having CAD, prior to confirmation by more precise diagnostic modalities and invasive angiography. The results from our study have important clinical implications for the diagnosis and management of CAD. The use of LV GLS and central aortic pulse pressure as complementary diagnostic markers offers a more comprehensive approach to risk stratification. Stiff aorta and LV GLS can be used to identify patients at higher risk of CAD who may benefit from early intervention. The integration of these markers into clinical practice could improve the accuracy of risk stratification and reduce the number of unnecessary invasive procedures.

There is previous evidence that the myocardial work index (pressure-strain loop) plays an important role in the detection and prognosis of ischemic heart disease. In patients with non-ST-segment elevation myocardial infarction, a segmentally reduced global work index can predict an occluded coronary artery, even when ejection fraction and global longitudinal strain are preserved [22]. Additionally, in patients with single-vessel coronary artery disease at rest, myocardial work has been shown to be more sensitive than LV GLS in detecting subclinical coronary disease, with all myocardial work parameters reduced in the segmental region supplied by the occluded artery [23,24]. Although our study did not include measurements of the myocardial work index in predicting physiologically significant coronary artery disease (CAD), our results align with these findings—demonstrating that LV GLS alone has limited diagnostic utility, while the ischemic Bull's-eye pattern and aortic stiffness provide incremental diagnostic value in identifying physiologically significant CAD.

Our study has several limitations. Firstly, it is a retrospective cohort study, and as such, missing data and unintentional biases may have occurred in certain situations. However, we made every effort to ensure accurate data collection and excluded patients with incomplete data from the analysis. Second, our results did not demonstrate promising outcomes regarding the LV GLS method's ability to reliably predict physiologically significant CAD. This may reflect the true diagnostic limitations of this method, as it was not able to predict significant CAD reliably. However, as previously mentioned, when using the ischemic Bull's-eye pattern derived from the LV GLS test, we observed high sensitivity, and a favorable diagnostic odds ratio. While the current evidence supports the utility of ischemic Bull's-eye pattern derived from LV GLS and central aortic pulse pressure in CAD diagnosis, further studies are needed to validate these findings in diverse populations and to establish standardized thresholds for clinical use.

## 6 Conclusion

The study underscores the potential of ischemic Bull's-eye pattern derived from LV GLS and central aortic pulse pressure as emerging predictive markers for physiologically significant CAD. While LV GLS alone has limited diagnostic utility, the ischemic Bull's-eye pattern derived from LV GLS and central aortic pulse pressure provide incremental diagnostic value. The integration of these markers with clinical risk factors and advanced imaging techniques offers a more comprehensive approach to CAD diagnosis and risk stratification. Future research should focus on optimizing these diagnostic tools and translating them into clinical practice to improve patient outcomes.

## CRediT authorship contribution statement

**Pratya Rawangban:** Writing – review & editing, Writing – original draft, Visualization, Validation, Supervision, Software, Resources, Project administration, Methodology, Investigation, Funding acquisition, Formal analysis, Data curation, Conceptualization. **Anusith Tunhasiriwet:** Writing – review & editing, Visualization, Validation, Supervision, Software, Resources, Project administration, Methodology, Investigation, Funding acquisition, Formal analysis, Data curation, Conceptualization. **Rawish Wimolwattanaphan:** Project administration, Methodology, Investigation, Data curation. **Chanwit Wuttichaipradit:** Writing – review & editing, Supervision, Conceptualization. **Piyoros Lertsanguansinchai:** Writing – review & editing, Writing – original draft, Visualization, Validation, Supervision, Software, Resources, Project administration, Methodology, Investigation, Funding acquisition, Formal analysis, Data curation, Conceptualization.
